# A model protocol for the cryopreservation and recovery of motile lizard sperm using the phosphodiesterase inhibitor caffeine

**DOI:** 10.1093/conphys/coaa044

**Published:** 2020-06-22

**Authors:** Lachlan Campbell, Shenae L Cafe, Rose Upton, J Sean Doody, Brett Nixon, John Clulow, Simon Clulow

**Affiliations:** 1 School of Environmental and Life Sciences, University of Newcastle, Callaghan, New South Wales 2308, Australia; 2 Department of Biological Sciences, Southeastern Louisiana University, Hammond, LA 70402, USA; 3 Department of Biological Sciences, University of South Florida, St. Petersburg, FL 33701, USA; 4 Department of Biological Sciences, Macquarie University, Sydney, New South Wales 2109 Australia

**Keywords:** Assisted reproduction, caffeine, cryoprotectant, dimethyl sulfoxide, genome storage, glycerol, lizard, reptile, sperm motility, spermatozoa

## Abstract

Reproductive technologies such as genome storage and assisted reproduction have a significant role to play in ending or reversing species extinctions. However, such technologies for non-model organisms (i.e. non-mammalian species) are poorly developed. This is particularly true for the reptiles, in which there is a dearth of successful protocols for cryopreserving reptile spermatozoa, despite limited attempts. We investigated sperm cryopreservation in the Australian lizard *Varanus panoptes* with the objective of addressing the unmet need for an optimized cryopreservation protocol for the spermatozoa of squamate reptiles. We tested the efficacy of two cryoprotectants [dimethyl sulfoxide (DMSO) and glycerol] as well supplementation with a phosphodiesterase inhibitor (caffeine) to promote post-thaw motility. For cryopreservation, sperm were cooled in straws suspended in liquid nitrogen vapour for 5 minutes (approximately −135°C), before being plunged into liquid nitrogen (approximately −196°C), and later thawed in a water bath at 35°C. Samples were incubated post-thaw for 10 minutes in the presence or absence of 10 mM of caffeine. Both cryoprotectant type and concentration significantly affected percent sperm motility pre-freezing, with DMSO being less cytotoxic than glycerol and motility decreasing at higher concentrations of both cryoprotectant types. While cold shock did not significantly affect sperm motility, both cryoprotectant type and concentration did significantly impact the motility of post-thawed spermatozoa. Thus, mid-range concentrations (10% v/v) of DMSO and glycerol yielded a greater post-thaw motility compared with 5 and 20% v/v, while DMSO proved superior to glycerol. The addition of caffeine resulted in a significant recovery of post-thaw motility for both cryoprotectants, with higher rates of motility being associated with higher cryoprotectant concentrations. These protocols provide a significant step forward for *in situ* and *ex situ* management of threatened reptiles and add to recent evidence that reptilian sperm may have the full range of phosphorylation-mediated cellular mechanisms associated with capacitation, motility and metabolic regulation found in mammalian sperm.

## Introduction

The collection and storage of genomes through sperm cryopreservation, combined with associated assisted reproductive technologies (ARTs) for restoring animals and genes using the genetic material, could play a large role in threatened species conservation (Browne *et al.*, 2011; Clulow and Clulow, 2016; Howard *et al.*, 2015). Despite the potential to apply these technologies to all vertebrate taxa, reptiles are a relatively neglected taxon, with few successful sperm cryopreservation protocols developed for this group despite some attempts and typically much lower rates of successful post-thaw recovery (Browne*, et al.*, 2011; Clulow and Clulow, 2016; Molinia *et al.*, 2010). This is important because up to 25% of reptilian species are threatened with extinction globally and a further 25% are classed as data deficient (Böhm *et al.*, 2013). Indeed, some reptile orders such as the Testudines and Crocodilia may be as high as 50% threatened with extinction globally (Böhm*, et al.*, 2013; Grigg and Kirshner, 2015).

Given the limited number of reports and species investigated across the reptiles, whose major lineages have evolved very different reproductive systems, there is a strong imperative to understand general principles and shared aspects of sperm cryopreservation protocols in one or more model reptile species. Ideally, these studies would serve as a prelude for the development of optimized protocols within and between reptilian groups. Lizards are one of the most diverse reptile groups, yet sadly, of the known approximately 6100 lizard species globally, the International Union for Conservation of Nature (IUCN) has classified at least one-third as threatened with extinction (Böhm*, et al.*, 2013; Gibbons *et al.*, 2000). Given their significance and diversity within squamate reptiles, lizards are an appropriate group to focus on for the development of an optimized sperm cryopreservation protocol, notwithstanding the success reported for one species (Young *et al.*, 2017). One such lizard that is currently suffering large population declines is the yellow-spotted monitor (*Varanus panoptes*), an Australian species that has been severely impacted by the presence of the invasive cane toad (*Rhinella marina*) (Doody *et al.*, 2009; Doody *et al.*, 2014; Doody *et al.*, 2017). Affected populations decline through increased mortality rates resulting from lethal toxic ingestion when the toad is consumed as prey (Doody*, et al.*, 2009; Doody*, et al.*, 2017; Ujvari and Madsen, 2009). Since its introduction to Australia in 1935 (Doody*, et al.*, 2019; Lever, 2006), the cane toad has caused population crashes of up to 97% in *V. panoptes* (Doody*, et al.*, 2009) as well as extirpations of this and at least one other monitor species in some parts of northern Australia (Doody*, et al.*, 2017), which in turn has resulted in rippling effects throughout ecosystems via trophic cascades (Doody, *et al.*, 2017; Doody *et al.*, 2015). *Varanus panoptes* is thus an ideal model species for the development of sperm cryostorage and ARTs, because it is abundant ahead of the toad front for conducting experiments, but is threatened behind the invasion vanguard and exerts a disproportionately large influence over its ecosystem, thus warranting urgent conservation attention.

Sperm cryopreservation involves a series of steps that must be optimized to minimize the cell damage associated with deep freezing (Dinnyes *et al.*, 2007). Dimethyl sulfoxide (DMSO) and glycerol are perhaps the two most commonly investigated cryoprotectants for the spermatozoa of wildlife species, although the relative success of each often varies depending on the species (Comizzoli *et al.*, 2012). Cryoprotective agents (CPAs) also become increasingly toxic as the concentration increases (Best, 2015), which can result from non-specific toxicity via water molecule interference with the cell membrane or specific toxicity derived from the CPA type and concentration (Fahy, 1986; Fahy, 2010). It is important to understand the interplay of these factors in order to develop appropriate conditions for sperm cryopreservation.

In addition to optimizing cryoprotectant, media and cooling rates as a part of the freeze-thaw protocol, other factors may aid in increasing the viability of sperm and thus improve their motility characteristics in preparation for ARTs such as *in vitro* fertilization (IVF) or artificial insemination. For example, the addition of the phosphodiesterase inhibitor caffeine has been shown to increase intracellular levels of the second messenger, cyclic-adenosine monophosphate (cAMP) (Pukazhenthi *et al.*, 2005; Saragusty, 2012), resulting in a consequential increase in forward progressive motility in unfrozen epididymal sperm of the lizard, *Lacerta vivipara* (Depeiges and Dacheux, 1985). In a similar context, cryopreserved mammalian spermatozoa have also been shown to display elevated levels of post-thaw motility following incubation in caffeine (Mbizvo *et al.*, 1993; Rota *et al.*, 2018; Schill *et al.*, 1979; Stachecki *et al.*, 1994). The addition of caffeine could thus potentially be a novel and simple means by which to stimulate post-thaw motility of lizard sperm, although to the best of our knowledge this principle has never been tested. Therefore, optimizing cryopreservation protocols requires attention to the separate components of cryoprotectant, media and cooling/thawing rates, but also may be enhanced by additional post-thaw treatments.

The objective of the present study was to develop an optimized sperm-freezing protocol in the model lizard *V. panoptes* and thus further our understanding of how to freeze reptile sperm and enhance their subsequent post-thaw motility. Specifically, we aimed to determine the following: (1) cytotoxicity of two potential cryoprotectants at various concentrations pre-freeze, that might offset beneficial effects of protecting against freeze damage; (2) whether reptile sperm are sensitive to cold shock elicited in response to rapid-cooling protocols; (3) which cryoprotectant and concentration provides the best protection for freezing the spermatozoa; and (4) whether post-thaw sperm motility could be improved by the addition of the phosphodiesterase inhibitor caffeine, thus potentially providing a novel method for optimizing reptile spermatozoa freeze-thaw protocols.

## Materials and methods

### Collection of animals, reproductive tracts and spermatozoa

*Varanus panoptes* males (n = 11) were collected from Fitzroy Crossing, Western Australia (−18.192410 °S, 125.566893 °E) over two breeding seasons (November to April) in 2015 (n = 5) and 2017 (n = 6). Animals were collected using cage traps (810 × 254 × 305 mm; Havahart, PA, USA) baited with kangaroo tails, checked twice daily. Animals were euthanized within 2 days of capture by blunt cranial displacement followed by immediate severing of the spinal cord. Immediately post-euthanasia, each animal was carefully dissected for the removal of both reproductive tracts. The testes and male genital ducts of lizards are intra-abdominal and paired with both tracts contributing to the production and storage of spermatozoa (left and right testes are of similar size) (Rheubert *et al.*, 2014). The major extra-testicular ducts are the epididymis and the ductus deferens and these are the primary sites of post-testicular sperm maturation and storage. The epididymis is regarded as the primary storage organ for sperm in lizards (de la Cruz *et al.*, 2014; Rheubert*, et al.*, 2014), although in *V. panoptes*, the ductus deferens is larger and holds more sperm than the epididymis during the reproductive season (unpublished data). Spermatozoa for this study were collected directly from the ductus deferens.

Upon dissection, the tracts and testes were immersed in Dulbecco’s phosphate-buffered saline (PBS; Ca^++^, Mg^++^ free) (Sigma-Aldrich, St. Louis, MO, USA) to prevent desiccation. The vas deferens of both tracts were then macerated in a 60 × 15 mm petri dish containing 1 ml PBS by making several incisions along its entire length. Following maceration, each dish was allowed to stand for 2 minutes to release the sperm from the tract. Excess tissue was removed from each dish to create the final sperm suspension for experiments. All animal experiments and procedures were conducted with approval of the University of Newcastle Animal Care and Ethics Committee (ethics authorization A-2016-601) and Western Australia scientific permit 08-001546-2.

### Cytotoxic effects of cryoprotectant type and concentration on sperm motility

The spermatozoa of four individuals collected during November 2015 were used to assess the cytotoxic effects of cryoprotectants on lizard sperm. Two to four sub-samples from each individual (14 in total) were incubated at room temperature (25°C) for 5 hours in PBS (Ca^++^, Mg^++^ free) with or without DMSO or glycerol (Univar USA Inc., Redmond, WA, USA) at concentrations of either 5, 10 or 20% v/v. After the 5-hour incubation period, the percentage motile sperm in each sample was assessed in duplicate under a light microscope with phase optics (Nikon Instruments, Melville, NY) at 400× magnification. At least 200 spermatozoa were assessed from a minimum of three randomly selected fields of view and the experiment repeated four times for each individual (one individual repeated twice).

### Effect of rapid cold shock

The spermatozoa of five individuals collected during November 2015 were assessed for potential susceptibility to cold shock as a prelude to later determination of optimal cooling rates for sperm cryopreservation. Three to four samples (5 μl) of sperm macerates in PBS (Ca^++^, Mg^++^ free) from each animal were placed on a glass microscope slide without a cover slip and incubated for 90 seconds at either room temperature (~25°C) or at −20°C in a standard freezer. Following cooling, each sample was incubated at room temperature for 2 minutes before the percentage of motile spermatozoa was assessed as described above.

### Effect of cryoprotectant type, cryoprotectant concentration during cryopreservation and post-thaw addition of caffeine on recovery of sperm motility

**Figure 1:**
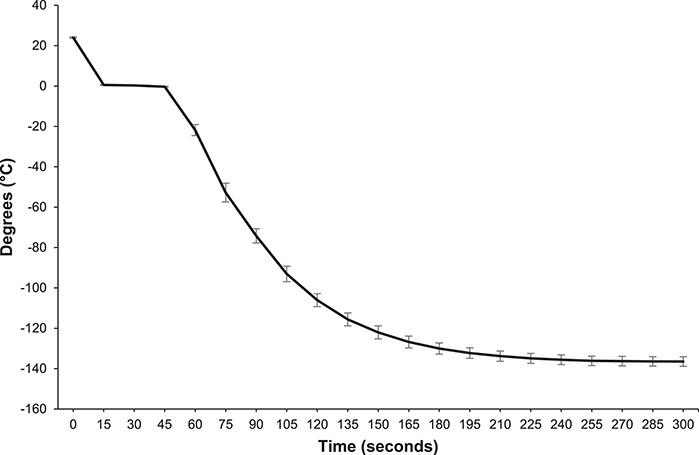
Cooling rate of water suspended 5 cm above LN_2_, immersed in vapour and cooled over 5 minutes. The data are presented as raw means ±1 standard error of the mean (n = 4 repetitions).

Sperm macerates from five animals collected during April 2017 (n = 2) and December 2017 (n = 3) were used to test the effects of freezing and thawing on sperm motility in varying concentrations of two cryoprotectants in PBS (Ca^++^, Mg^++^ free) media, followed by post-thaw addition of caffeine. Sperm macerate sub-samples from the two animals collected in April 2017 were diluted 1:1 in either DMSO or glycerol in PBS to achieve a final concentration of 5% v/v (n = 20 straws), 10% v/v (n = 24 straws) or 20% v/v (n = 24 straws). Individual sperm macerate sub-samples (50 μl) were loaded into 250 μl straws in triplicate and were cooled rapidly at a mean rate of approximately −32.1°C/minute (Fig. 1) by suspending the straws horizontally at 5 cm above liquid nitrogen in a foam ice box (Johnston *et al.*, 2017). The cooling rate was determined by measuring the internal temperature of a 250-μl straw loaded with 50 μl of water and frozen following the same methods for the sperm-loaded straws. Temperature was measured using a thermocouple inserted into cryostraws containing distilled water and was repeated four times. After 5 minutes, the straws were plunged directly into liquid nitrogen and transferred to a liquid nitrogen dewar (Taylor-Wharton, Theodore, Alabama) for storage. The samples were subsequently thawed after ~48 hours of storage by immersing the straws into a ~500 ml of water bath at 35°C for ~1 minute. Each thawed straw was diluted 1:1 in PBS (Ca^++^, Mg^++^ free) with or without the presence of 20 mM caffeine (Sigma-Aldrich, St. Louis, MO, USA) to achieve a final caffeine concentration of 10 mM. Sperm macerate sub-samples from the three animals collected in December 2017 were subjected to the same freeze-thaw protocols above, but only using DMSO at a concentration of 10% v/v to serve as a direct comparison of the effect of season at that cryoprotectant concentration (end of wet season vs start of following wet season). The percentage of motile sperm was then scored for all treatments as described above. Additionally, the percentage of live and dead sperm cells was determined through fluorescence microscopy by diluting samples 1:1 with propidium iodide (stock concentration, 1.5 mM) (Sigma-Aldrich, St. Louis, MO, USA) and immediately assessing following a 10-second incubation at room temperature. The first 100 cells were counted and determined as live (no fluorescence) or dead (red fluorescence).

**Figure 2:**
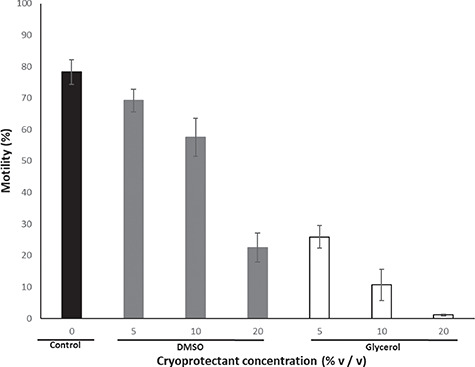
The effect of cryoprotectant type and concentration on percent sperm motility in unfrozen samples incubated for 5 hours at room temperature (~25°C). The data are presented as raw mean percentage ± 1 standard error of the mean.

### Statistical analysis

All data were analysed by the non-parametric Kruskal–Wallis test using JMP Statistical Software (version 11, SAS Institute, Cary, NC, USA), with individual comparisons between treatments determined through Wilcoxon each pair analysis. Analyses were conducted on data derived from replicated sperm samples (straws) within individuals for each treatment.

## Results

### Animal morphometrics

The mean snout-to-vent length of captured animals was 647 ± 112 mm, while the average individual mass was 3120 ± 810 g. The mean total testes mass was 8.89 ± 3.17 g. The mean concentration of sperm in a 1 ml sample was 2.64 × 10^6^ ± 5.6 × 10^5^ cells/ml. All animals had motile spermatozoa in the vas deferens except for one individual, which was removed from the study.

### Cytotoxic effects of cryoprotectant type and concentration assessed from sperm motility in unfrozen samples

The mean sperm concentration was 2.69 × 10^6^ ± 1.21 × 10^6^ cells/ml, which did not affect the sperm motility for PBS samples (R^2^ = 0.135). Both cryoprotectant type (χ^2^ = 37.45, df = 1, *P* < 0.001) and cryoprotectant concentration (χ^2^ = 23.97, df = 2, *P* < 0.001) significantly affected percent sperm motility when present for 5 hours in unfrozen samples (Fig. 2). DMSO was less cytotoxic than glycerol at equivalent concentrations, with percent motility decreasing as cryoprotectant concentration increased for both cryoprotectants (Fig. 2). There was no significant difference in sperm motility when incubated at the highest DMSO concentration (20% v/v) compared with the lowest glycerol concentration (5% v/v; χ^2^ = 1.27, df = 1, *P* = 0.26). The highest motility maintained in the presence of a cryoprotectant was in 5% v/v DMSO, with a mean of 69.2 ± 4.2% motile sperm when incubated at room temperature, which was not statistically different to the PBS only (no cryoprotectant) control (78.3 ± 4%; χ^2^ = 3.21, df = 1, *P* = 0.07). Sperm incubated in 10% v/v DMSO also retained high percent motility (57.5 ± 6.1%) comparable to the 5% v/v treatment (χ^2^ = 2.37, df = 1, *P* = 0.124), but all other cryoprotectant media markedly reduced percent sperm motility when incubated at room temperature (Fig. 2).

**Figure 3:**
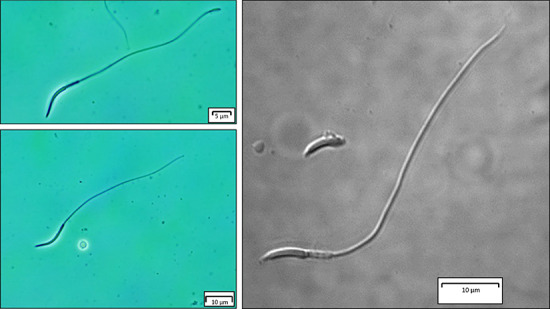
Light and confocal microscopy images of normal *V. panoptes* sperm following cryopreservation*.* Sperm were frozen in 10% v/v DMSO.

**Figure 4:**
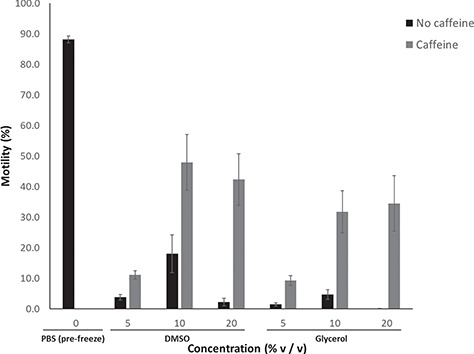
The effect of cryoprotectant type and concentration during cryopreservation and the addition of 10 mM caffeine post-thaw on percent sperm motility assessed after thawing of samples. Data are presented as raw means ±1 standard error of the mean.

**Figure 5:**
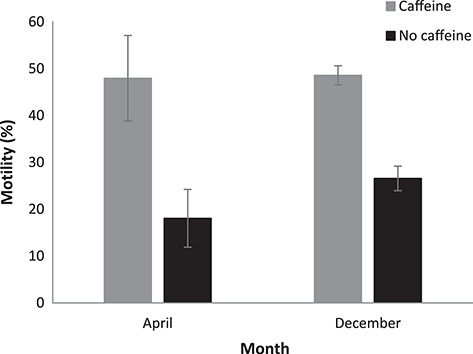
Comparison of post-thaw sperm motility between animals captured during April (n = 2) and December (n = 3) for samples frozen with 10% v/v DMSO, with or without the presence of caffeine. Data are presented as raw means ±1 standard error of the mean (derived from 12 repetitions per animal).

### Effect of rapid cold shock

Exposure to a rapid temperature decline (cold shock) did not affect mean sperm motility (83.1% ± 2.5) compared with sperm exposed to room temperature only (82.9% ± 3.2) (χ^2^ = 0.241, df = 1, *P* = 0.624).

### Effect of freezing and thawing on sperm motility assessed post-thaw in the presence or absence of caffeine.

#### Samples without post-thaw addition of caffeine

**Figure 6:**
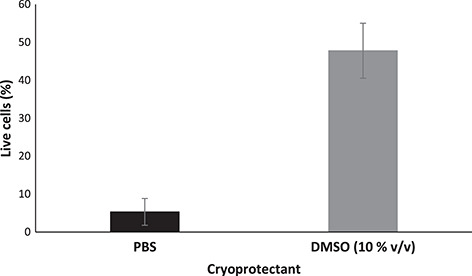
Comparison between the percentage of live cells following sperm cryopreservation (n = 4) with 10% v/v DMSO. Data are presented as raw means ±1 standard error of the mean.

The sperm concentration for both individuals was 2.96 × 10^6^ and 2.64 × 10^6^ cells/ml. Both cryoprotectant type (χ^2^ = 6.89, df = 1, *P* = 0.009) and concentration (χ^2^ = 17.98, df = 2, *P* < 0.001) significantly affected the percent motility of post-thawed spermatozoa, with the mid-range concentration (10% v/v) yielding the highest post-thaw recovery for both cryoprotectants and DMSO yielding higher post-thaw motility than glycerol (Figs. 3 and 4 ). However, even the best protocol, utilizing 10% v/v DMSO, yielded a mean post-thaw motility of only 18.1 ± 6.2%; a marked reduction compared to the 57.5 ± 6.1% motility observed for sperm incubated in 10% v/v DMSO at room temperature for 5 hours, or to unfrozen sperm in PBS (88.2 ± 1.2%) (Figs 2 and 4). The percentage of intact plasma membranes also differed between sperm frozen in PBS only (5.3 ± 3.5%) compared with sperm frozen in 10% v/v DMSO (47.8 ± 7.2%) (χ^2^ = 5.4, df = 1, *P* = 0.02) (Fig. 6).

#### Samples with post-thaw addition of caffeine

The addition of caffeine to the sperm samples post-thaw significantly increased the percent motility of sperm for all cryoprotectant protocols (χ^2^ = 58.68, df = 1, *P* < 0.001; Fig. 4). The positive effect of caffeine was further influenced by the concentration of both DMSO and glycerol cryoprotectants, with higher concentrations resulting in a significantly higher percentage of motile sperm (χ^2^ = 12.79, df = 2, *P* = 0.002; Fig. 4). The highest recovery after the post-thaw addition of caffeine occurred in 10% v/v and 20% v/v DMSO, resulting in post-thaw motility of 48 ± 9.1% and 42.3 ± 8.4%, respectively; comparable to the percent motility observed at equivalent cryoprotectant concentrations pre-freeze (Figs 2 and 4).

#### Comparison of post-thaw samples (10% v/v DMSO) between seasons (April vs. December 2017)

Samples of sperm from animals collected at the end of one breeding season (April 2017; n = 2), did not differ in their post-thaw motility between both caffeine and non-caffeine samples when compared to animals collected at the start of the following breeding season (December 2017; n = 3) (χ^2^ = 1.42, df = 1, *P* = 0.233; Fig. 5). Mean post-thaw motility for caffeine samples was 47.96 ± 6.4% in April and 48.6 ± 5.9% in December 2017, while samples without caffeine had a mean post-thaw motility of 18.06 ± 4.7% and 26.58 ± 4.3% for April and December 2017, respectively.

## Discussion

The main outcomes for our study are two-fold: (1) we demonstrated the capacity to yield high levels of post-thaw motile sperm in the lizard *V. panoptes* and (2) post-thaw incubation in caffeine containing media resulted in a marked and statistically significant increase in percent motility, suggesting reversible metabolic cryobiological effects rather than irreversible structural damage during freeze-thaw protocols. As far as we are aware, our sperm cryopreservation and recovery protocol for this lizard species using the novel approach of post-thaw incubation in caffeine has produced the highest post-thaw recovery of motile spermatozoa for any reptile reported to date (Browne*, et al.*, 2011; Clulow and Clulow, 2016; Young*, et al.*, 2017). Importantly, this study has also highlighted the different types of damage that may occur within spermatozoa as a consequence of their cryopreservation, i.e. reversible loss of motility, probably due to reduction in metabolic substrates or co-factors (Barkay *et al.*, 1977; Mbizvo*, et al.*, 1993), versus irreversible structural damage (Holt, 2000a; Holt, 2000b).

We found the greatest recovery of sperm motility after cryopreservation was produced with a protocol involving a combination of 10% v/v DMSO in PBS (Ca^++^, Mg^++^ free) and a cooling rate of −32.1°C/min for 5 minutes before plunging into LN_2_, followed by a 10-minute post-thaw incubation in 10 mM of caffeine with PBS, resulting in 48% post-thaw motility of cryopreserved samples (compared to ~88.2% of motility in unfrozen samples). This was also repeated in a separate set of animals (n = 3) where there was no difference in post-thaw motility, thus demonstrating the repeatability of the protocol (Fig. 4). Additionally, the interaction between high CPA concentration and caffeine on increased sperm motility showed that the effects of cryopreservation in permeating CPAs are reversible following thawing. However, this was not the case for low CPA concentrations (5% v/v) illustrating that the reversibility is a concentration-dependent phenomenon. This may reflect a reduced capacity of cryoprotectants to protect against irreversible structural damage at lower CPA concentrations, in contrast to higher concentrations that may protect against structural damage even if they are exerting some cytotoxic effects (Elliott *et al.*, 2017; Gao and Critser, 2000; Holt, 2000a).

Cryodamage is not limited to the sperm plasma membrane, but can also affect mitochondrial activity resulting in reduced ATP production and metabolism required to sustain motility, partially mediated through loss of cAMP and other cytoplasmic factors to the external environment (Elliott*, et al.*, 2017; O'connell *et al.*, 2002). Phosphodiesterase inhibitors such as caffeine are known to increase intracellular levels of cAMP in sperm (Lardy *et al.*, 1971), and cAMP is a major mediator of sperm motility through control of downstream phosphorylation events via its role in regulation of kinase activity. Indeed, recent work has shown that reptilian sperm may have the full range of phosphorylation-mediated cellular mechanisms associated with capacitation, motility and metabolic regulation found in mammalian sperm (Nixon *et al.*, 2016; Nixon *et al.*, 2019), and the action of caffeine on *V. panoptes* sperm in the current study suggests that restoration of activity in these pathways may be occurring through increased levels of cAMP in post-thaw sperm induced by the addition of caffeine.

The evidence here suggests there is a complex interaction between the cytotoxicity of cryoprotectants and their protective effects against cryodamage analogous to the trade-offs in the two factor (cooling rate/dehydration versus osmotic damage) cryoinjury model of Mazur (Mazur *et al.*, 1972). DMSO was a more successful CPA than glycerol for both room temperature and frozen-thawed sperm in terms of maintaining motility, which is similar to the report for the Argentinian black and white tegu, *Salvator merianae* (Young*, et al.*, 2017). In contrast, the only other accounts of reptilian sperm cryopreservation featuring a comparison of DMSO and glycerol were in the saltwater crocodile (*Crocodylus porosus*), where it was found that 5% v/v glycerol was the most effective cryoprotectant (Johnston*, et al.*, 2017), and an abstract describing the behaviour of rattle snake sperm that also suggested glycerol was a more effective cryoprotectant (Zacariotti *et al.*, 2011). Although there have been sporadic and taxonomically dispersed attempts at reptile sperm cryopreservation, the percentage of viable spermatozoa recovered post-thaw is variable and often lower than reported for other vertebrate taxa (Clulow and Clulow, 2016). For example, snake semen has been cryopreserved with ~30% post-thaw recovery (Millar and Watson, 2001), whereas the best reported outcome for crocodilian semen cryopreservation ranges from 9.3–14.2% mean post-thaw motility (Johnston*, et al.*, 2017). In what appears to be the only report of lizard sperm cryopreservation, the mean recovery of post-thaw motility as a percentage of the initial fresh motility was 47.8% (Young*, et al.*, 2017), among the highest reported recovery rates for reptilian sperm. Our post-thaw motility for samples frozen in 10% v/v DMSO when compared to the initial sperm motility was 54.4%, which is greater than any previous reported levels of post-thaw motility for a lizard species. Although these differences could be attributed to species-specific effects or differences between protocols (i.e. freeze-rate, media type, thawing rate, etc.), our results demonstrate the applicability of sperm cryopreservation in reptile species. While it appears that glycerol is a less effective CPA than DMSO for *V. panoptes*, this should be tested for other lizard species before generalized protocols advocating DMSO as the rule are put forward.

Our findings provide significant progress for the development of a model cryopreservation protocol for lizards, building on the recent report by Young*, et al.* (2017) with Argentinian black and white tegu sperm, with a protocol that yields high levels of post-thaw motile sperm. Future research should aim to test how applicable the current media conditions and sperm handling procedures optimized for *V. panoptes* are to other species of lizards, as well as protocols for other lineages of reptiles that are more phylogenetically removed such as the turtles and crocodilians. Studies in other groups of vertebrates suggest that ART protocols are not always transferable between long-divided lineages (Clulow and Clulow, 2016; Clulow *et al.*, 2018; 2019), and hence it is important to test the current method for *V. panoptes* across multiple lineages within the Reptilia.

## References

[ref1] Barkay J, Zuckerman H, Sklan D, Gordon S (1977) Effect of caffeine on increasing the motility of frozen human sperm. Fertil Steril 28: 175–177. 832730 10.1016/s0015-0282(16)42378-2

[ref2] Best BP (2015) Cryoprotectant toxicity: facts, issues, and questions. Rejuvenation Res 18: 422–436. 25826677 10.1089/rej.2014.1656 PMC4620521

[ref3] Böhm M, Collen B, Baillie JE, Bowles P, Chanson J, Cox N, Hammerson G, Hoffmann M, Livingstone SR, Ram M (2013) The conservation status of the world’s reptiles. Biol Conserv 157: 372–385.

[ref4] Browne R, Li H, Robertson H, Uteshev V, Shishova N, McGinnity D, Nofs S, Figiel C, Mansour N, Lloyd R (2011) Reptile and amphibian conservation through gene banking and other reproduction technologies. Russ J Herpet 18: 165–174.

[ref5] Clulow J, Clulow S (2016) Cryopreservation and other assisted reproductive technologies for the conservation of threatened amphibians and reptiles: bringing the arts up to speed. Reprod Fertil Dev 28: 1116–1132. 10.1071/RD15466 27246622

[ref6] Clulow J, Pomering M, Herbert D, Upton R, Calatayud N, Clulow S, Mahony MJ, Trudeau VL (2018) Differential success in obtaining gametes between male and female Australian temperate frogs by hormonal induction: a review. Gen Comp Endocrinol 265: 141–148. 29859744 10.1016/j.ygcen.2018.05.032

[ref7] Clulow J, Upton R, Trudeau VL, Clulow S (2019) Amphibian assisted reproductive technologies: moving from technology to application In Comizzoli P, Brown JL, Holt WV, eds, Reproductive Sciences in Animal Conservation. Springer, Cham, pp. 413–463. 10.1007/978-3-030-23633-5_14 31471805

[ref8] Comizzoli P, Songsasen N, Hagedorn M, Wildt D (2012) Comparative cryobiological traits and requirements for gametes and gonadal tissues collected from wildlife species. Theriogenology 78: 1666–1681. 22704386 10.1016/j.theriogenology.2012.04.008

[ref9] de la Cruz FRM, Manríquez NL, Ríos EA, Ibargüengoytía N (2014) Male reproductive cycles in lizards In Rheubert JL, Siegel DS, Trauth SE, eds, Reproductive Biology and Phylogeny of Lizards and Tuatara. CRC Press: Taylor & Francis Group, Boca Raton, pp. 314–351.

[ref10] Depeiges A, Dacheux J (1985) Acquisition of sperm motility and its maintenance during storage in the lizard, *Lacerta vivipara*. J Reprod Fertil 74: 23–27. 4020769 10.1530/jrf.0.0740023

[ref11] Dinnyes A, Liu J, Nedambale T (2007) Novel gamete storage. Reprod Fertil Dev 19: 719–731. 17714626 10.1071/rd07035

[ref12] Doody J, Green B, Rhind D, Castellano C, Sims R, Robinson T (2009) Population-level declines in Australian predators caused by an invasive species. Animal Conserv 12: 46–53.

[ref13] Doody JS, Mayes P, Clulow S, Rhind D, Green B, Castellano CM, D’Amore D, Mchenry C (2014) Impacts of the invasive cane toad on aquatic reptiles in a highly modified ecosystem: the importance of replicating impact studies. Biol invasions 16: 2303–2309.

[ref14] Doody JS, Rhind D, Green B, Castellano C, McHenry C, Clulow S (2017) Chronic effects of an invasive species on an animal community. Ecology 98: 2093–2101. 28477376 10.1002/ecy.1889

[ref15] Doody JS, Soanes R, Castellano CM, Rhind D, Green B, McHenry CR, Clulow S (2015) Invasive toads shift predator–prey densities in animal communities by removing top predators. Ecology 96: 2544–2554. 26594710 10.1890/14-1332.1

[ref16] Doody JS, McHenry C, Letnic M, Everitt C, Sawyer G, Clulow S (2019) Forecasting the spatiotemporal pattern of the cane toad invasion into North-Western Australia. Wildlife Res 45: 718–725.

[ref17] Elliott GD, Wang S, Fuller BJ (2017) Cryoprotectants: a review of the actions and applications of cryoprotective solutes that modulate cell recovery from ultra-low temperatures. Cryobiology 76: 74–91. 28428046 10.1016/j.cryobiol.2017.04.004

[ref18] Fahy GM (1986) The relevance of cryoprotectant “toxicity” to cryobiology. Cryobiology 23: 1–13. 3956226 10.1016/0011-2240(86)90013-1

[ref19] Fahy GM (2010) Cryoprotectant toxicity neutralization. Cryobiology 60: S45–S53. 19501081 10.1016/j.cryobiol.2009.05.005

[ref20] Gao D, Critser J (2000) Mechanisms of cryoinjury in living cells. ILAR J 41: 187–196. 11123179 10.1093/ilar.41.4.187

[ref21] Gibbons JW, Scott DE, Ryan TJ, Buhlmann KA, Tuberville TD, Metts BS, Greene JL, Mills T, Leiden Y, Poppy S (2000) The global decline of reptiles, déjà vu amphibians reptile species are declining on a global scale. Six significant threats to reptile populations are habitat loss and degradation, introduced invasive species, environmental pollution, disease, unsustainable use, and global climate change. BioScience 50: 653–666.

[ref22] Grigg G, Kirshner D (2015) Biology and Evolution of Crocodylians. CSIRO Publishing, Ithica.

[ref23] Holt W (2000a) Basic aspects of frozen storage of semen. Animal Reprod Sci 62: 3–22. 10.1016/s0378-4320(00)00152-4 10924818

[ref24] Holt W (2000b) Fundamental aspects of sperm cryobiology: the importance of species and individual differences. Theriogenology 53: 47–58. 10735061 10.1016/s0093-691x(99)00239-3

[ref25] Howard J, Lynch C, Santymire R, Marinari P, Wildt D (2015) Recovery of gene diversity using long-term cryopreserved spermatozoa and artificial insemination in the endangered black-footed ferret. Animal Conserv 19: 102–111.

[ref26] Johnston S, Qualischefski E, Cooper J, McLeod R, Lever J, Nixon B, Anderson A, Hobbs R, Gosálvez J, López-Fernández C (2017) Cryopreservation of saltwater crocodile (*Crocodylus porosus*) spermatozoa. Reprod Fertil Dev 29: 2235–2244. 28356183 10.1071/RD16511

[ref27] Lardy HA, Garbers DL, Lust W, First NL (1971) Effects of phosphodiesterase inhibitors and cyclic nucleotides on sperm respiration and motility. Biochemistry 10: 1825–1831.

[ref28] Lever C (2006) The Cane Toad: The History and Ecology of a Successful Colonist. Springer, West Yorkshire.

[ref29] Mazur P, Leibo S, Chu E (1972) A two-factor hypothesis of freezing injury: evidence from chinese hamster tissue-culture cells. Exp Cell Res 71: 345–355. 5045639 10.1016/0014-4827(72)90303-5

[ref30] Mbizvo MT, Johnston RC, Baker GH (1993) The effect of the motility stimulants, caffeine, pentoxifylline, and 2-deoxyadenosine on hyperactivation of cryopreserved human sperm. Fertil Steril 59: 1112–1117. 8486183

[ref31] Millar J, Watson P (2001) Cryopreservation of gametes and embryos in reptiles and amphibians In Watson PF, Holt WV, eds, Cryobanking the Genetic Resource: Wildlife Conservation for the Future. Taylor & Francis, London, pp. 171–178.

[ref32] Molinia FC, Bell T, Norbury G, Cree A, Gleeson DM (2010) Assisted breeding of skinks or how to teach a lizard old tricks. Herpetol Conserv Biol 5: 311–319.

[ref33] Nixon B, Anderson AL, Smith ND, McLeod R, Johnston SD (2016) The Australian saltwater crocodile (*Crocodylus porosus*) provides evidence that the capacitation of spermatozoa may extend beyond the mammalian lineage. Proc R Soc B Biol Sci 283: 20160495. 10.1098/rspb.2016.0495 PMC4874718 27147099

[ref34] Nixon B, Johnston SD, Skerrett-Byrne DA, Anderson AL, Stanger SJ, Bromfield EG, Martin JH, Hansbro PM, Dun MD (2019) Modification of crocodile spermatozoa refutes the tenet that post-testicular sperm maturation is restricted to mammals.. Mol Cell Proteomics 18: S59–S76. 30072580 10.1074/mcp.RA118.000904 PMC6427239

[ref35] O'connell M, McClure N, Lewis S (2002) The effects of cryopreservation on sperm morphology, motility and mitochondrial function. Hum Reprod 17: 704–709. 11870124 10.1093/humrep/17.3.704

[ref36] Pukazhenthi B, Comizzoli P, Travis AJ, Wildt DE (2005) Applications of emerging technologies to the study and conservation of threatened and endangered species. Reprod Fertil Dev 18: 77–90. 10.1071/rd05117 16478605

[ref37] Rheubert JL, Sever DM, Siegel DS, Trauth SE (2014) Male reproductive anatomy: the gonadoducts, sexual segment of the kidney and cloaca In Rheubert JL, Siegel DS, Trauth SE, eds, Reproductive Biology and Phylogeny of Lizards and Tuatara. CRC Press: Taylor & Francis Group, Boca Raton, pp. 253–301.

[ref38] Rota A, Sabatini C, Przybył A, Ciaramelli A, Panzani D, Camillo F (2018) Post-thaw addition of caffeine and/or pentoxifylline affect differently motility characteristics of horse and donkey cryopreserved spermatozoa. J Equine Vet Sci 66: 85–86. 10.1016/j.jevs.2019.01.003 31002091

[ref39] Saragusty J (2012) Genome Banking for Vertebrates Wildlife Conservation. INTECH Open Access Publisher.

[ref40] Schill W, Pritsch W, Preissler G (1979) Effect of caffeine and kallikrein on cryo-preserved human spermatozoa. Int J Fertil 24: 27–32. 37176

[ref41] Stachecki JJ, Ginsburg KA, Armant DR (1994) Stimulation of cryopreserved epididymal spermatozoa of the domestic cat using the motility stimulants caffeine, pentoxifylline, and 2 ‘-deoxyadenosine. J Androl 15: 157–164. 8056639

[ref42] Ujvari B, Madsen T (2009) Increased mortality of naive varanid lizards after the invasion of non-native can toads (*Bufo marinus*). Herpetol Conserv Biol 4: 248–251.

[ref43] Young C, Ravida N, Curtis M, Mazzotti F, Durrant B (2017) Development of a sperm cryopreservation protocol for the Argentine black and white tegu (*Tupinambis merianae*). Theriogenology 87: 55–63. 27639519 10.1016/j.theriogenology.2016.08.006

[ref44] Zacariotti R, Guimarães M, Jensen T, Durrant B (2011) Cryopreservation of snake semen: are we frozen in time? Reprod Fertil Dev 24: 171–171.

